# Postoperative lumbar spinal stenosis after intertransverse fusion with granules of hydroxyapatite: a case report

**DOI:** 10.1186/1746-1596-7-153

**Published:** 2012-11-07

**Authors:** Gen Inoue, Seiji Ohtori, Tomoyuki Ozawa, Toshinori Ito, Morihiro Higashi, Kazuyo Yamauchi, Sumihisa Orita, Junichi Nakamura, Tomoaki Toyone, Masashi Takaso, Kazuhisa Takahashi

**Affiliations:** 1 Department of Orthopaedic Surgery, Kitasato University, School of Medicine, 1-15-1 Kitasato, Minami-ku, Sagamihara, Kanagawa, 252-0374, Japan; 2 Department of Orthopaedic Surgery, Graduate School of Medicine, Chiba University, 1-8-1 Inohana, Chuo-ku, Chiba, 260-8670, Japan; 3 Department of Orthopaedic Surgery, Teikyo University Chiba Medical Center, 3426-3 Anesaki, Ichihara, Chiba, 299-0111, Japan; 4 Department of Pathology, Saitama Medical Center, Saitama Medical University, 1981 Kamoda, Kawagoe, Saitama, 350-8550, Japan

**Keywords:** Nonunion, Lumbar spine, Intertransverse fusion, Hydroxyapatite

## Abstract

In the present case of postoperative lumbar spinal stenosis after non-instrumented intertransverse fusion with granules of hydroxyapatite (HA), bone union was not completed and the patient felt the recurrence of his symptoms within two years. We performed re-decompression with fusion, and in hematoxylin and eosin staining of HA granulation harvested during revision surgery, fibrous tissue with hyaline degeneration surrounded the cavity where the HA had existed. Multinuclear giant cells and lymphocytes infiltrated some parts of the marginal layer of the cavity, and no obvious bony bridge had regenerated from autologous bone. No tartrate-resistant acid phosphate (TRAP) -positive osteoclasts could be seen in the new bone, suggesting that the activity of osteoclasts in the new bone decreased during the seven years after the primary surgery.

**Virtual slides:**

The virtual slide(s) for this article can be found here: http://www.diagnosticpathology.diagnomx.eu/vs/3483360258050263

## Case report

**Figure 1:**
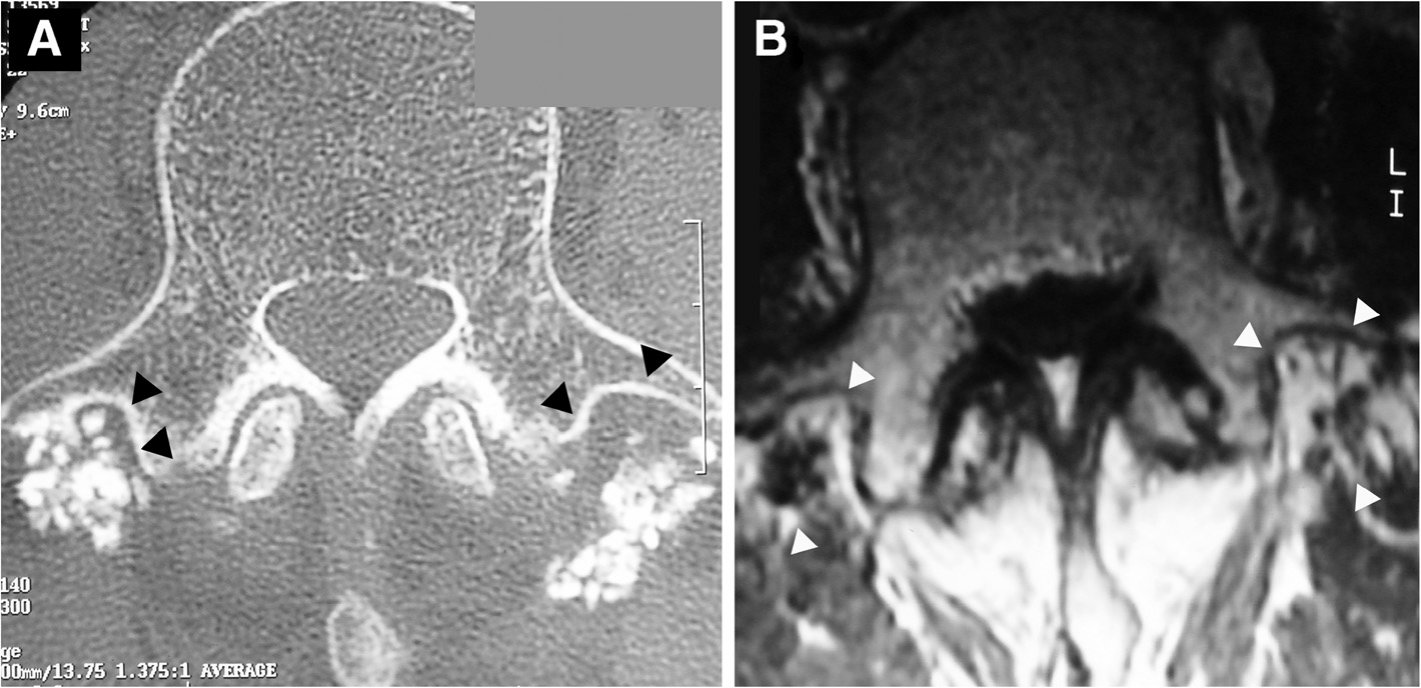
**Preoperative imagings A; Computed tomography of L4 showing scant bone formation around the granulation tissue and few bony intertransverse bridges between the transverse process and HA.** Because of intervening fibrous tissue, contact between HA and the vertebrae is limited (arrowheads). **B**; Gadolinium-enhanced magnetic resonance imaging (MRI) of L4. Enhancement was observed around transplanted HA (arrowheads), suggesting failure of the posterolateral fusion.

A 76-year-old man with lumbar spinal stenosis was admitted to our hospital with low back pain and bilateral sciatica. For his low back and bilateral leg pain, he had undergone L4/L5 non-instrumented intertransverse fusion with granules of HA for lumbar spinal stenosis in another hospital seven years before. After surgery, his complaint disappeared completely, however, after two years, he once again felt low back pain and increasing bilateral sciatica. On admission, he showed intermittent claudication after 20 meters, and lateral roentgenograms showed instability, suggesting failure of the attempted posterolateral intertransverse fusion. Computed tomography showed that hardly any bone had formed around the HA and no bony intertransverse bridge existed between the transverse process and HA (black arrow heads; Figure 1A). Magnetic resonance imaging (MRI) of the T2 wedged axial plane revealed a high intensity area between the mass of hydroxyapatite and the transverse process. Enhancement with gadolinium was observed around the transplanted HA, suggesting failure of the intertransverse fusion (Figure 1B). Decompression and instrumented intertransverse fusion with autologous iliac bone for L4/L5 were performed after removal of the HA granulation. Both sides of the granules of HA that formed an *en bloc* mass were resected obtusely and evaluated histologically.

## Histological findings

**Figure 2:**
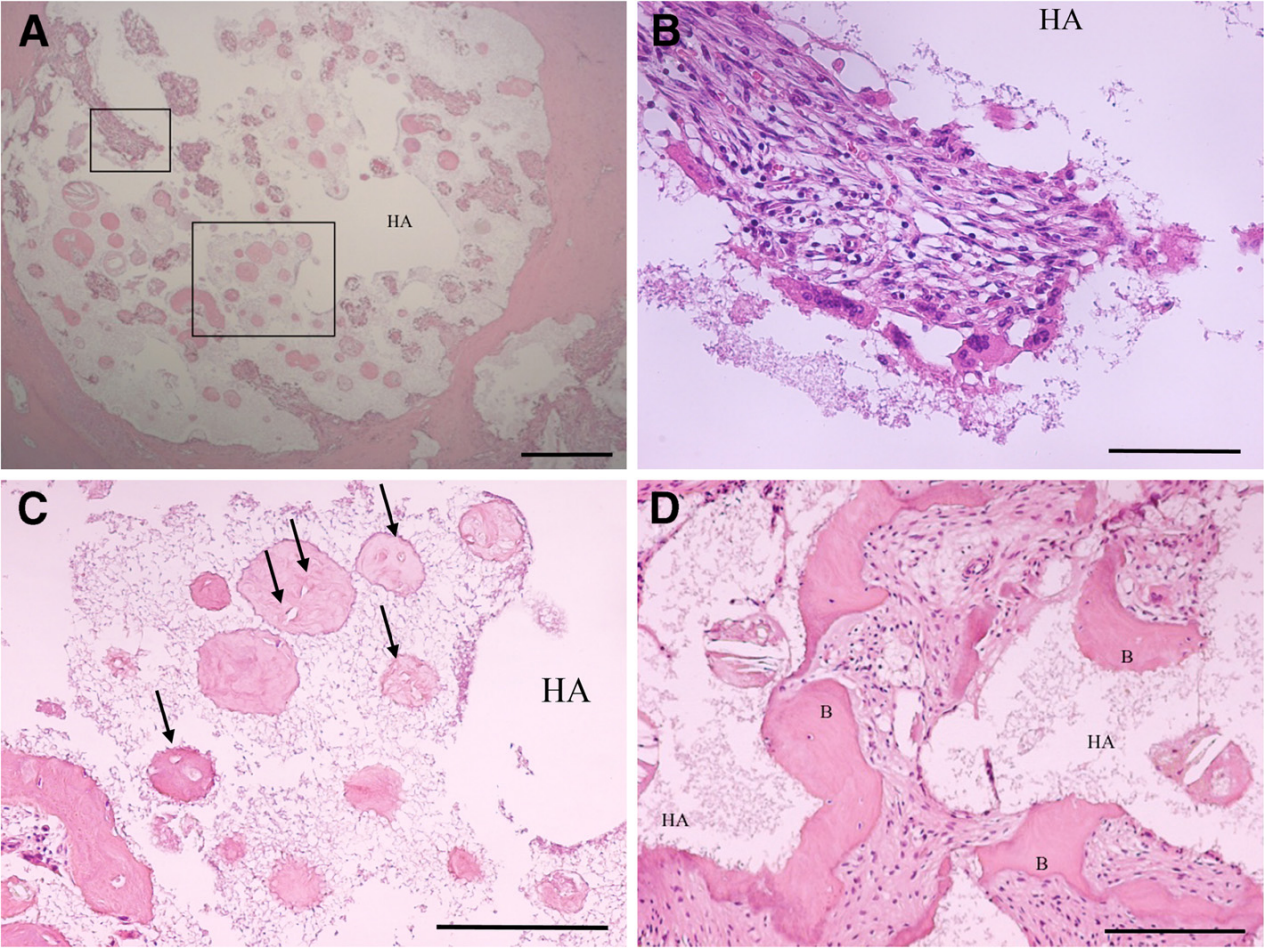
**Hematoxylin and eosin staining of a sagittal section of the resected mass. A**; Fibrous tissue with hyaline degeneration surrounded the cavity where HA existed. (magnification, ×25; scale bar, 500 μm). **B**; A higher magnification of the boxed area in Figure A. In some parts of the marginal layer of the cavity, multinuclear giant cells and lymphocytes were observed, partially forming a foreign body granuloma (magnification, ×200; scale bar, 100 μm). **C**; A higher magnification of the boxed area in Figure A. Scant new bone formation is found in the HA cavity, or juxtaposed with fibrous tissue. Some new bone was necrotic. Black arrows indicate necrotic bone without osteocytes (magnification, ×100; scale bar, 200 μm). **D**; Further observation of new bone formation juxtaposed with fibrous tissue. Osteocytes were present in the new bone, but bone marrow formation was not observed (magnification, ×100; scale bar, 200 μm). 'B', New bone.

**Figure 3:**
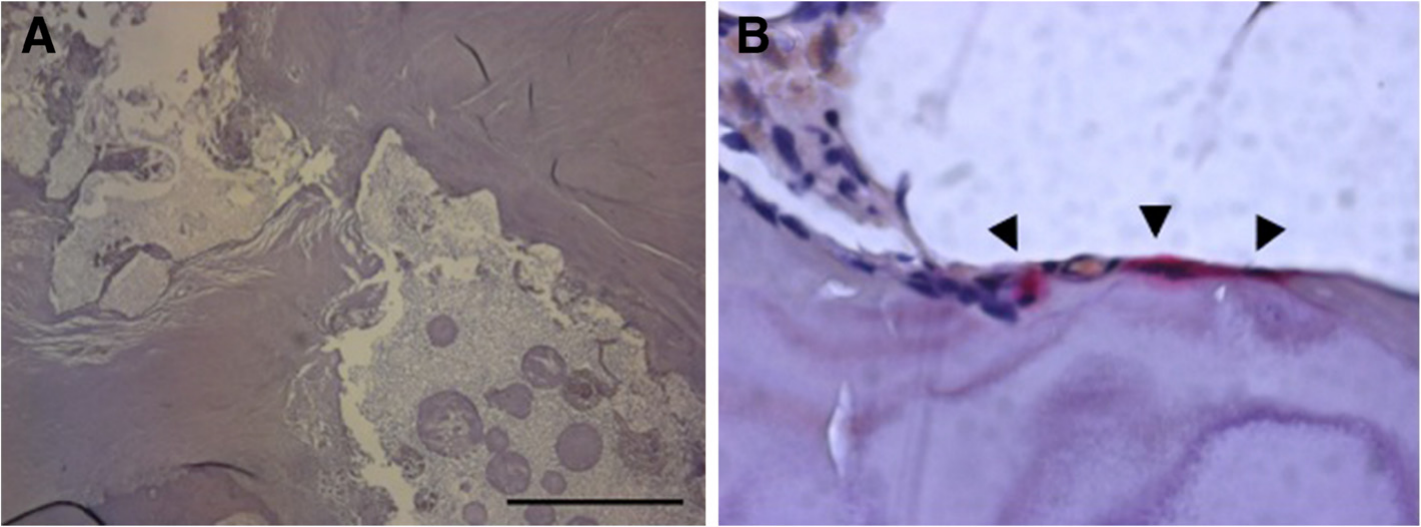
**Tartrate-resistant acid phosphatase (TRAP) staining of a sagittal section of the resected mass. A**; No TRAP-positive osteoclasts could be seen around the new bone or the cavity (scale bar, 500μm). **B**; Bone tip harvested from a spinous process during the corrective surgery was used as a positive control. Some TRAP-positive osteoclasts were observed (arrow heads).

In hematoxylin and eosin staining of the sagittal section of the resected mass, fibrous tissue with hyaline degeneration surrounded the cavity where the HA had existed. Multinuclear giant cells and lymphocytes infiltrated some parts of the marginal layer of the cavity, and partially formed foreign-body granulomas. New bone formation was observed in a limited area in the cavity where the HA had been, or just beside the fibrous tissue. Most of this new bone was necrotic. Bone marrow formation was not seen in any sections (Figure 2A–D). In tartrate-resistant acid phosphate (TRAP) staining, TRAP-positive osteoclasts were not seen in the new bone in spite of the presence of TRAP-positive osteoclasts in the section of the spinous process that was harvested during the surgery (Figure 3). These findings suggest that the activity of osteoclasts decreased during the seven years after the primary surgery and that insufficient formation of new bone around the HA granules resulted in failure of the intertransverse fusion.

## Discussion

Since the first report in 1953, lumbar posterolateral intertransverse fusion has been most commonly performed for patients with lumbar instability and has been reported to achieve successful union and clinical outcomes [1-5]. Recently, hydroxyapatite (HA) has been used as a substitute for autologous bone graft, and there have been no detailed reports of failure of intertransverse fusion with HA [6,7].

There have been few reports of long-term follow-up in cases of intertransverse fusion with granules of HA. It is reported that macrophages responding to HA particles are capable of osteoclast differentiation, and new bone formation occurs between and within HA granules when bone union is successful [8]. In the present case, histological findings showed that formation of osteoclasts did not continue and that activity of these cells ceased within seven years. This was accompanied by insufficient bone formation from failure of coupled remodeling. On the other hand, in some parts of the marginal layer of the cavity, multinuclear giant cells and lymphocytes were observed, partially forming a foreign body granuloma. These cells are usually observed with HA, but few inflammatory reaction was observed in the whole tissue which was resected, indicating patient’s pain was not induced by inflammation of the transplanted tissue, but might be induced by another unstable structures including the intervertebral discs or facet joints [9-11]. Motomiya et al [12]. reported the bony bridge which is achieved within autologous bone graft may be formed more easily and earlier than bone ingrowth to HA, but no obvious bony bridge which regenerated from autologous bone was seen in the present case. The exact pathomechanism for nonunion is unclear, but for spinal fusion, factors in the host (*e.g*., nutrition, age, smoking, mechanical stress), in biology (type or quality of bone graft, fusion site preparation, internal fixation), or in postoperative care (activity level, external immobilization) are reported to affect the union success rate in both human and animal studies [13,14]. These complex factors might influence bone formation and may have induced failure of the intertransverse fusion.

Also, preparation of fusion site might be insufficient in this case. More careful preparation of the soft tissue bed or bone surface onto which the graft material is placed might be necessary, the importance of which is indicated in previous reports [15,16].

Recently, many factors (*e.g.*, growth factor, bone morphogenetic protein) that stimulate bone formation have been investigated as a substitute for autologous bone graft, but these kinds of bone substitutes are still prohibited for spine surgery in Japan [17,18]. So, most bone substitutes that are used in Japan are HA and β-tricalcium phosphate, which can provide a scaffold for new bone formation. Because fusion status is critical to achieve good post-operative results, research in failed cases is very important [19]. Further understanding of the causes and mechanisms of nonunion are important to achieve higher union rates and to avoid poor clinical results in patients after surgery.

## Consent

Written informed consent was obtained from the patients for publication of this case report and any accompanying images.

## Competing interests

The author(s) declared no conflicts of interest with respect to the authorship and/or publication of this article.

## Authors’ contributions

GI and MH carried out the histochemical explorations. SO, TO, TI and KT participated in the surgery and the design of the study. JN, TT and MT conceived the study and have been involved in the literature search. GI, KY and SO drafted the manuscript. All authors read and approved the final manuscript.

## Funding

The author(s) received no financial support for the research and/or authorship of this article.
